# Melatonin as a Novel Drug to Improve Cardiac Function and Quality of Life in Heart Failure Patients: A Systematic Review and Meta‐Analysis

**DOI:** 10.1002/clc.70107

**Published:** 2025-03-03

**Authors:** Abolfazl Sam Daliri, Nima Goudarzi, Arshia Harati, Kourosh Kabir

**Affiliations:** ^1^ Medical Student, School of Medicine Alborz University of Medical Sciences Karaj Iran; ^2^ Department of Community Medicine School of Medicine Iran University of Medical Sciences Tehran Iran

**Keywords:** cardiac potency, ejection fraction, heart failure, melatonin, meta‐analysis, NT‐Pro BNP, NYHA function classes, Quality of life, stroke volume, systematic review

## Abstract

**Background:**

Heart failure as an advanced cardiac disease has a high incidence and prevalence in all societies nowadays. Many drugs and treatment methods have been discovered for improving heart failure patients' conditions till now in this way melatonin therapy is one of the less‐known methods rarely used by clinicians.

**Methods:**

To investigate the positive effect of melatonin on heart failure development, we conducted a systematic review and meta‐analysis by searching valid databases with keywords based on the protocol. Based on the eligible criteria, four articles were selected for data synthesis and analysis after scanning the title and/or abstract and reading full‐text.

**Results:**

As a result of analysis, increasing ejection fraction (Mean difference: 2.39 [−1.82, 6.59] *p* = 0.27), *NYHA* (New York Heart Association Functional Class) (Odds ratio: 4.84 [1.00, 23.44] *p* = 0.05), and significant elevation of quality of life (Mean difference: −5.95 [−9.54, −2.35] *p* = 0.001) were observed. As the effect of melatonin, fatigue, and *NT‐Pro BNP* were reduced but on the contrary sleep quality, appetite, and *FMD* (Flow‐Mediated Dilation) significantly increased.

**Conclusion:**

Thus, melatonin, by increasing psychologic parameters and cardiac potency, could be advised as a novel drug for treatment and palliating heart failure patients.

## Graphical abstract

To investigate the effect of melatonin on heart failure development, we conducted a systematic review and meta‐analysis. We searched several databases, including Scopus, PubMed, Web of Science, and Cochrane, using keywords based on the protocol. Initially, 8013 manuscripts were included. After screening and reading full texts based on eligible criteria, this number was reduced to 4 articles for data synthesis and analysis.

The analysis result indicated an increase in ejection fraction (Mean difference: 2.39 [−1.82, 6.59] *p* = 0.27), an improvement in *NYHA* (New York Heart Association Functional Class) (Odds ratio: 4.84 [1.00, 23.44] *p* = 0.05), and a significant enhancement in quality of life (Mean difference: −5.95 [−9.54, −2.35] *p* = 0.001). Therefore, we concluded that melatonin could significantly improve the quality of life for heart failure patients.

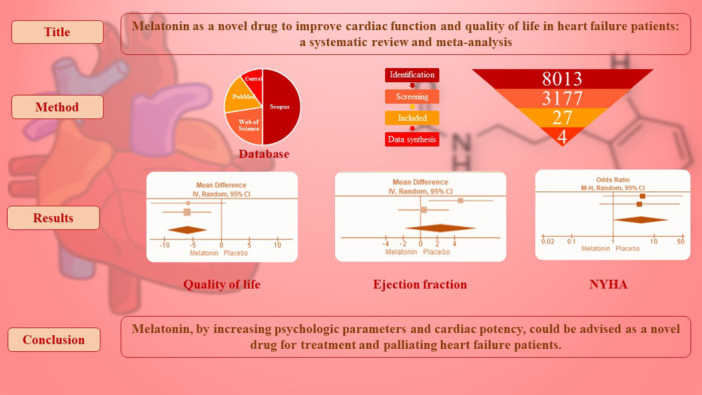

**Abbreviations**
BP: blood pressure
CI: confidence interval
EF: ejection fraction
FMD: flow‐mediated dilation
HF: heart failure
HFrEF: heart failure with reduced ejection fraction
LVEF: left ventricular ejection fraction
MLHFQ: the minnesota living with heart failure questionnaire
NT‐Pro BNP: N‐Terminal pro‐B‐type Natriuretic Peptide
NYHA FC: New York Heart Association Functional Class
QoL: Quality of Life
RCT: randomized controlled trial
RevMan: review manager
SD: standard deviation
SV: stroke volume

## 1 Introduction

*HF* (Heart Failure) is an advanced heart disease in which cardiac pumping function is significantly reduced in advanced conditions. This disease has a high prevalence in the world in this way 64.3 million people were estimated to have suffered in 2017, although its prevalence, incidence, and survival are variable in different countries [1, 2]. Despite lifetime risk of developing *HF* in men and women being almost the same throughout life, its occurrence increases by old age in women more than in men [3, 4]. *HF* can occur as a result of myocardial ischemia, high blood pressure, infection, etc and the risk can be increased by many different parameters such as smoking, diabetes, obesity, and hypertension [5, 6, 7, 8, 9, 10, 11]. *HF* as a basic disease causes many secondary problems such as pulmonary edema, thromboembolism [11, 12, 13], impaired kidney or liver functions, respiratory distress, etc [14, 15, 16, 17]. However, there are many treatment methods to improve and palliate primary and advanced‐stage cardiac failure. Catheter ablation, gene therapy, hormone therapy, drug therapy, etc have been discovered till now, some are common methods but despite usefulness, other methods have been paid less attention [18, 19, 20]. Drug therapy as a classic method has significantly developed especially in recent years in this way many classes of drugs such as angiotensin‐converting enzyme inhibitors, Beta‐blockers, Mineralocorticoid receptor antagonists, Digoxin, and other stuff are used in the vast domain of *HF* patients [21]. Hormone therapy, as a less used method, can be used as a way of drug therapy for helping *HF* patients [19, 22, 23]. Melatonin therapy is a kind of hormone therapy in which melatonin or sleep hormone is used as a drug for cancer, myocardial infarction, depression, etc [24, 25, 26, 27]. Melatonin, an indolamine hormone, is secreted more by epiphysis and less by the bone marrow, retina, skin, and gastrointestinal tract [28, 29]. This hormone regulates sleep rhythm naturally; however, it can be used as a drug for regulating insomnia and other sleep problems [30]. Furthermore, melatonin by antioxidant features protects the body from free radicals [31, 32] so it could be used for conditions such as cancer and myocardial ischemia‐reperfusion injury, etc [24, 25]. With attention to sleep regulating, antioxidant, etc features of melatonin, it guesses that melatonin may be helpful for *HF* patients' *QoL* (Quality of Life) and cardiac potency. Thus, many studies have been performed to evaluate melatonin efficacy on psychological and cardiovascular parameters in *HF* patients [22, 23, 33, 34, 35]. However, the outcomes were invalid due to the low number of participants and scattered data in different studies; therefore, we conducted a systematic review and meta‐analysis based on all related *RCTs* (randomized controlled trial) to resolve the mentioned problems.

## 2 Methods

### 2.1 Search Strategy

**Figure 1:**
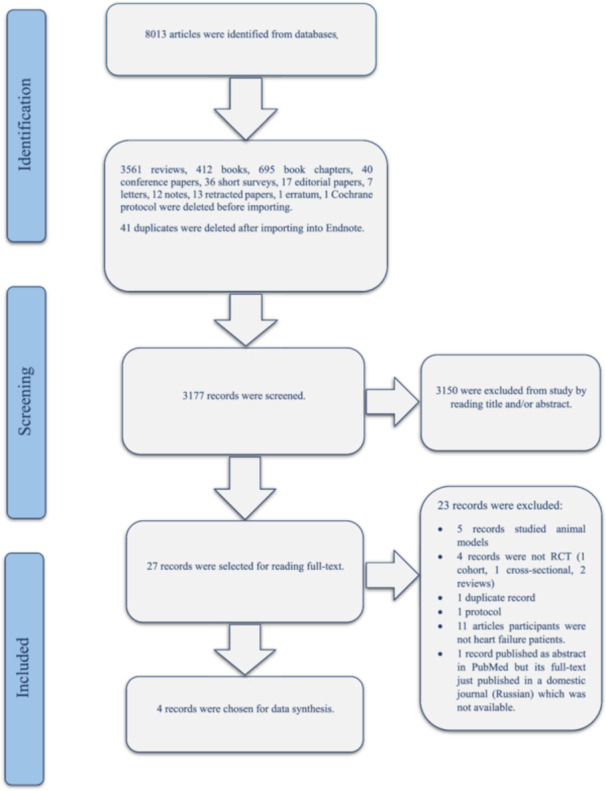
Flow chart of study selection.

We (ASD and NG) searched PubMed, Scopus, Web of Science, and Cochrane Library on November 28, 2023. Based on protocol, mesh, and synonyms of *HF*, melatonin and outcomes (*QoL*, *NT‐Pro BNP* (N‐terminal pro‐B‐type natriuretic peptide) and Stroke volume) were searched in the mentioned databases for all articles that investigate the effect of melatonin on the improvement of *QoL* and heart function in *HF* patients. As a result of searching all fields in databases, 8013 manuscripts were found; including original articles, reviews, books, letters, etc. (Figure 1).

### 2.2 Eligibility Criteria

We (NG and ASD) scanned the records that remained after the deletion of reviews, books, letters, duplicates, etc by reading the title and/or abstract, then differences between outcomes of scanning performed by NG and ASD; were found and solved by AH. Studies were selected for data synthesis and analysis exactly according to protocol.

The study protocol was designed based on PICOT framework, which includes participant, intervention, comparison, outcome, and type of study. Only *RCT* (randomized controlled trial) studies were selected for data synthesis and analysis. All eligible studies investigated the effect of oral melatonin tablets as an intervention compared to placebo tablets in heart failure patients classified as *NYHA* II and III. The main outcomes of the studies include psychological parameters (*QoL*), serum markers (*NT‐Pro BNP*), and cardiovascular parameters (Stroke volume and *NYHA*).

As a result, 27 articles remained for reading full‐text; Ultimately, four articles were selected for data synthesis, from which three articles were chosen for data analysis.

### 2.3 Data Extraction

Data from residual records were extracted by ASD and NG, then any differences were investigated and resolved by AH. Extracted data include the first author's name, year of publication, type of study, participant characteristics (age, gender, and sample size), type of comparison, outcome (Stroke volume, *QoL*, *NYHA*, and *NT‐Pro BNP*), and conclusion of the study.

### 2.4 Risk of Bias Assessment

The risk of bias for selected studies was independently evaluated through Review Manager software (Version 5.4.1 The Cochrane Collaboration, 2020) by AH and NG. Biases, which consist of random sequence generation, allocation concealment, blinding of participants and personnel, blinding of outcome assessment, incomplete outcome data, selective reporting, and other bias (follow‐up patients), were classified into three groups: low risk (green), high risk (red), unclear risk (yellow). Ultimately, any differences and disagreements are checked and resolved by ASD.

### 2.5 Data Analysis and Synthesis

Data from eligible studies were analyzed with Review Manager software (Version 5.4.1 The Cochrane Collaboration, 2020). Outcomes were classified into three categories: a) *NYHA* b) *EF* (Ejection Fraction) c) *QoL*. Comparison of these outcomes was performed by considering the mean difference with 95% *CI* for analyzing data of *QoL* and *EF*, and also odds ratio with 95% *CI* for *NYHA* which all results were reported as forest plot graphs. Furthermore, the random effect analysis model and inverse variance statistical method were used to decrease heterogeneity and variance between the selected studies. *P* and $I^{2}$ values were used to show the validity and homogeneity of the results and studies. Analyzing *EF* and *QoL* was performed by using mean and SD (standard deviation). However, in studies in which SD hadn't been reported, SD was measured by using start and end *CI* with *RevMan* (Review Manager) calculator.

## 3 Result

### 3.1 Literature Search

Scopus, PubMed, CENTRAL, and Web of Science databases were systemically searched with keywords (mesh and synonyms of (heart failure AND melatonin AND (*QoL* OR *SV* OR *NT‐Pro BNP*))) based on protocol. 8013 records were found from which, 3561 reviews, 695 book chapters, 412 books, 41 duplicates, 40 conference papers, 36 short surveys, 17 editorial papers, 13 retracted papers, 12 notes, 7 letters, 1 erratum, 1 Cochrane protocol were excluded before screening. 3177 records were scanned by reading title and/or abstract from which 3150 records were excluded and 27 records remained for full‐text screening. 23 manuscripts were excluded by full‐text reading because 5 records studied animal models, 4 records were not *RCT* (1 cohort, 1 cross‐sectional, 2 reviews), 1 duplicate record (published in double languages), 1 protocol, in 11 articles participants were not heart failure patients, 1 record published as abstract in PubMed but its full‐text just published in a domestic journal (Russian) which was not available. Ultimately, four articles were selected for data synthesis (*Jafari‐Vayghan 2022* [23], *Hoseini 2022* [33], *Hoseini 2021* [22], *and Garakyaraghi 2012* [34]) from which three articles were used for data analysis. Actually, *Hoseini 2021 and 2022* manuscripts were common *RCT* studies (same population) and outcomes were published in two separate articles*. EF* and *NYHA* classes as outcomes of *Hoseini 2022* and *Garakyaraghi 2012* and *QoL* as the outcome of *Jafari‐Vayghan 2022* and *Hoseini 2022* were separately analyzed.

### 3.2 Quality Assessment

**Figure 2:**
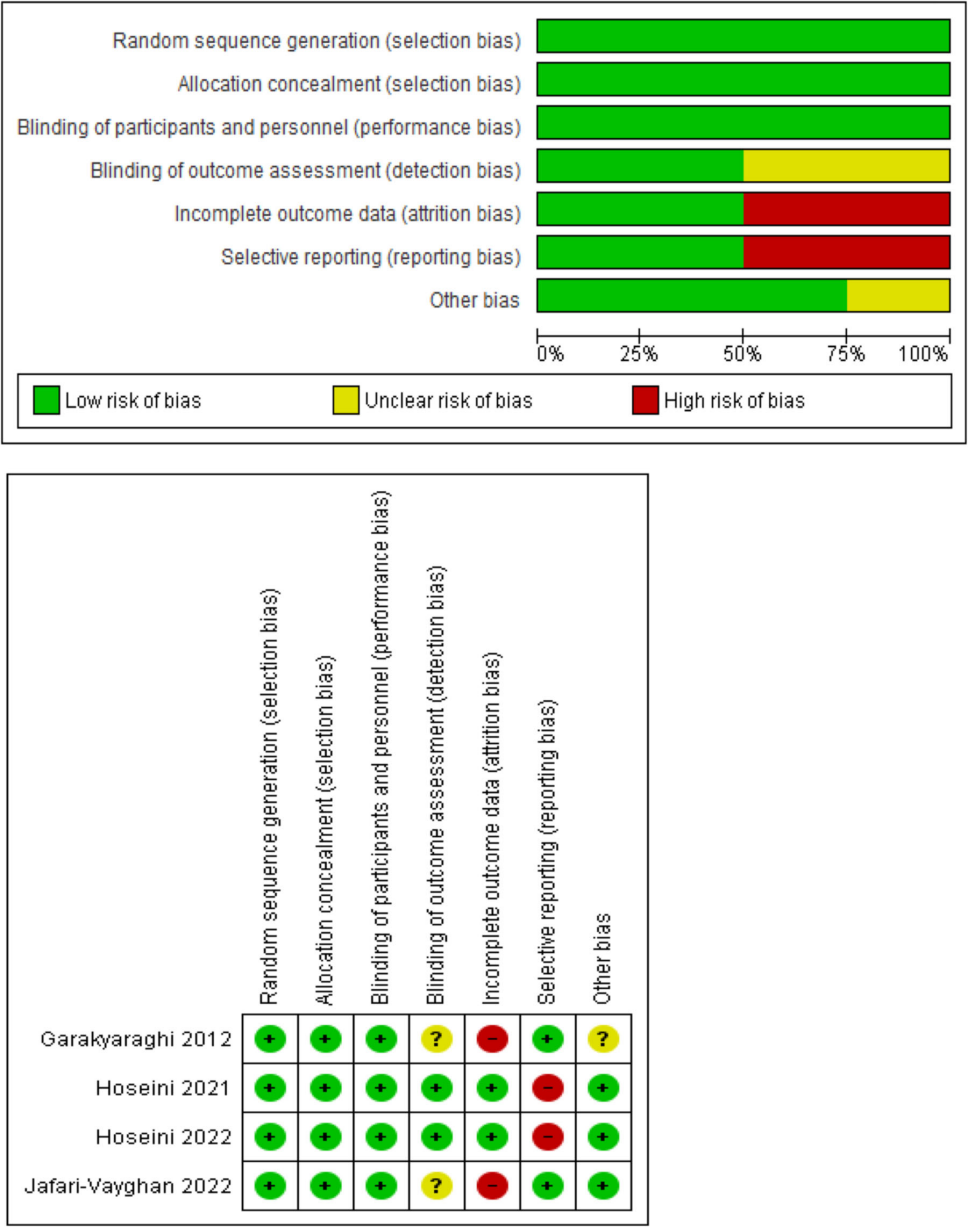
The overall and summary of risk of bias.

The quality assessment of the included studies was performed in the Cochrane scoring system by NG and AH. Disagreements and differences were resolved by discussion among the members. The overall risk of studies is shown in Figure 2. Selection and performance biases were completely low risk but 50% of detection, reporting, and attrition biases were unclear, high, and high risk, respectively. The other bias was low risk except for 25% which was unclear. The risk of bias for each study is demonstrated in Figure 2.

### 3.3 Literature Characteristics

#### 3.3.1 Participant

Four *RCT* studies were selected for systematic review in this way one was published in 2012 [34], one was published in 2021 [22], and two were published in 2022 [23, 33]. 166 people participated in the studies (132 men and 34 women) and the mean age of them ranged from 50.82 to 65.8. 83 and 76 people remained in melatonin and control groups, respectively after exclusion. All patients were categorized in II and III *NYHA* classification (Table 1).

**Table 1:** Study characteristics.

Number	Author, Year	Study design	Sample size (Melatonin/Control)	Gender (Male/Female)	Age (Melatonin/Control)	Type of intervention	Type of comparison
1	Garakyaraghi et al. [34]	RCT	23/16	27/12	63.6 (±6.6)/65.8 (±12.5)	3 mg/day for 8 weeks	Placebo (cellulose pills)
2	Hoseini et al. [22]	RCT	42/43	80/12^a^	62.7 (±10.3)/59.1 (±11.5)^a^	10 mg/day for 24 weeks	Placebo tablets
3	Hoseini et al. [33]	RCT	42/43	80/12^a^	63.5 (±22.89)/58.5 (±15.15)^a^	10 mg/day for 24 weeks	Placebo tablets
4	Jafari‐Vayghan et al. [23]	RCT	18/17	25/10	55.78 (±11.57)/50.82 (±11.22)	20 mg/day for 24 weeks	Placebo (cornstarch tablets)

Number	Outcome			Author conclusion
	*QoL* (Melatonin–Control)	*EF* (Melatonin–Control)	*NYHA* (Melatonin–Control)	
1	—	5.8 (±6.3) – 1.1 (±5.5)	6/23/1/16	The present study showed that melatonin may be an adjunctive option to improve *LVEF* (Left Ventricular Ejection Fraction) and functional class of patients with heart failure of different etiologies. However, larger studies would help to more clearly define its role in heart failure management.
2	—	—	—	Melatonin might be beneficial for the endothelial function in patients with *HFrEF* (Heart Failure with Reduced Ejection Fraction), and as a consequence, it could improve their outcome. Nevertheless, according to our results, this benefit in diabetic patients with *HFrEF* is debated and should be examined in a properly designed study.
3	22.2 (± 15.4) – 28 (± 15.59)	29.5 (± 7.7016) – 29.1 (±5.8488)	4/42/1/43	Melatonin might lower serum *NT‐Pro BNP* and improve disease‐specific health‐related quality of life in patients with *HFrEF*. Thus, it could be a valuable supplement for these patients. Further studies in subgroups of patients with *HF*, such as diabetic or hypertensive cardiomyopathic patients, and sensitive evaluation methods for cardiac function might provide new information in this regard.
4	4.89 (± 7.15) – 1.12 (± 5.74)	—	—	The findings of this study indicate that cosupplementation of *BCAAs* and melatonin for 8 weeks improved the *CC* outcomes such as *QoL*, fatigue status, and appetite in cachectic HF patients, but it could not affect *NRI* (nutritional risk index). Although separate supplementation with melatonin and *BCAAs* had positive effects on *QoL*, fatigue, and appetite of study participants, this study demonstrated that adding *BCAAs* to melatonin augmented these effects more than either of the 2 other supplements given alone.

a Data at the onset of study.

#### 3.3.2 Intervention

Oral melatonin was used for intervention groups (3, 10, and 20 mg/d for 8, 24, and 24 weeks, respectively), and placebo was used for control groups (cellulose pills and cornstarch tablets) (Table 1).

### 3.4 Data Synthesis

#### 3.4.1 Garakyaraghi 2012

*Garakyaraghi* et al. studied the effect of melatonin on left ventricular *EF* and *NYHA* changes in *HF* patients. Both parameters improved as a result of the intervention, in this way the baseline of *EF* in the intervention and control groups was 31.8 ± 7.8 and 34.1 ± 9.9, also after intervention, this parameter was 37.6 ± 7.1 and 35.3 ± 6.9 for the mentioned groups, respectively. During the trial process, the development of *EF* for melatonin and control groups was 5.8 ± 6.3 and 1.1 ± 5.5. Thus, the improvement of *EF* was more significant in the intervention group compared to control. Participants of the intervention group were classified in *NYHA* class II (20 patients) and III (3 patients), and the control group in *NYHA* class II (14 patients) and III (2 patients) before intervention. As the effect of melatonin therapy, improvement in 6 patients was observed (class I: 6 patients, class II: 14 patients, class III: 3 patients), the placebo effect also caused improvement in just a patient in the control group (class I: 1 patient, class II: 13 patients, class III: 2 patients).

#### 3.4.2 Hoseini 2021

*Hoseini* et al. measured the effect of melatonin therapy on *BP* (blood pressure) and *FMD* (flow‐mediated dilation) parameters in *HF* patients. Covariance analysis was used for adjusting the baseline parameters of the study, therefore the difference in end‐point data demonstrates the variation between intervention and control groups. Justified end‐point systolic/diastolic *BP* for melatonin and control groups were 121.7/76.1 and 116/72.4, respectively. Although the development of *BP* was seen, there is no valid correlation between melatonin therapy and improvement of *BP* (systolic *p* = 0.157 and diastolic *p* = 0.098). Significant elevation of mean *FMD*, as a prognostic parameter, was seen in nondiabetic *HF* patients (4.65% [1.14, 7.88], *p* = 0.006) but there is no significant change in mean *FMD* in diabetic *HF* patients (0.10% [−4.27, 4.06], *p* = 0.960).

#### 3.4.3 Hoseini 2022

The effect of melatonin on *NT‐Pro BNP*, *NYHA*, *EF* (serum and cardiovascular parameters), and *QoL* (for measuring the development of life conditions) was studied by *Hoseini* et al. As mentioned, in *Hoseini's* study covariance analysis was used. *NT‐Pro BNP* in intervention and comparison groups were 221.1 [148.9, 293.2] and 332.1 [253.5, 410.7] so cardiac stress was decreased as the effect of melatonin therapy (*p* = 0.044). Improvement of *NYHA* in four patients in the melatonin group and a patient in the control was observed, in this way *NYHA* class 4.42 [0.47, 41.31] times developed in intervention to control groups. However, *Hoseini* measured the *NYHA's* odds ratio, by considering the deterioration, 12.9 [1.6, 102.4] (*p* = 0.015) but we considered just the improvement of patients for adjusting to *Garakyaraghi's* study in data analysis. *EF*, according to the mean difference (0.40 [−2.51, 3.31]), doesn't have valid improvement. However, *QoL* development was observed but due to *p* ≥ 0.05, the outcome was invalid.

#### 3.4.4 Jafari‐Vayghan 2022

*Jafari‐Vayghan* et al. studied the effect of melatonin on *QoL* (*MLHFQ* (The Minnesota Living with Heart Failure questionnaire)), fatigue (*FSI* (Fatigue Symptom Inventory)), appetite (*SNAQ* (Simplified Nutritional Appetite Questionnaire)), and *NRI* (nutritional risk index) in cardiac cachexic *HF*. As the effect of melatonin *QoL* (*p* = 0.01), appetite (*p* = 0.004), fatigue (*p* = 0.002), and *NRI* (*p* = 0.015) status significantly were improved. *QoL* had two dimensions (physical and emotional dimensions); despite that, the physical dimension was significantly improved (*p* = 0.048) in the melatonin group, but any valid changes weren't seen in the emotional dimension (*p* = 0.495).

### 3.5 Data Analysis

#### 3.5.1 Effect of Melatonin on *QoL*

**Figure 3:**
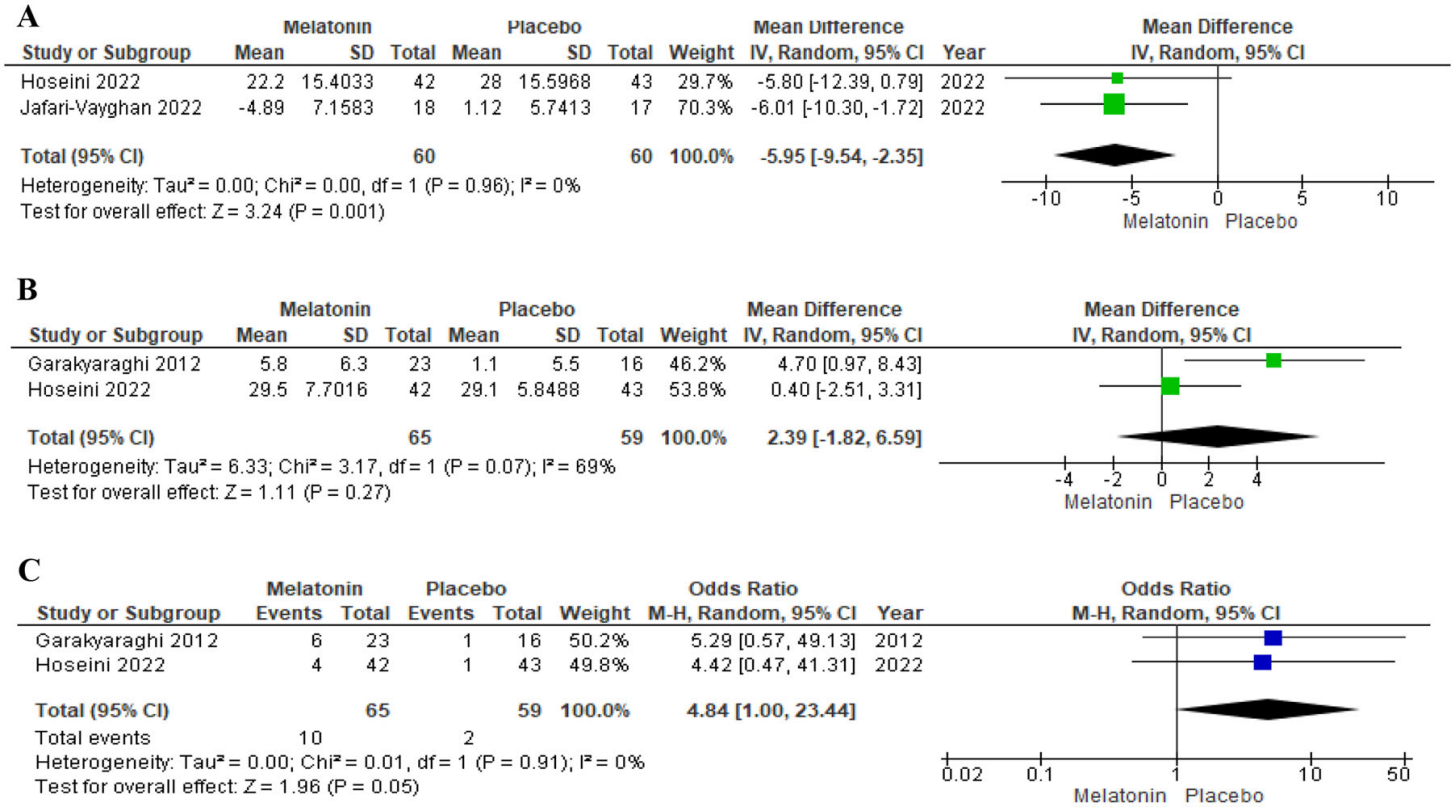
(A) Effect of melatonin on *QoL*. (B) Effect of melatonin on *EF*. (C) Effect of melatonin on *NYHA*.

As selected studies, two records investigated the effect of melatonin on *QoL* in *HF* patients. The results of studies were reported by mean difference and the validity was reported by 95% *CI*, *P*, and $I^{2}\text{values}.$ Both studies have demonstrated a positive effect of melatonin on *HF*, in this way −5.80 [−12.39, 0.79] improvement of *QoL* for *Hoseini 2022* and −6.01 [−10.30, −1.72] for *Jafari‐Vayghan 2022*. *Hoseini 2022* adjusted the baseline data of the study by using covariance analysis; therefore, the difference in the ultimate result shows the variation of outcomes (*QoL*, *EF*, etc). These studies used *MLHFQ* for evaluating *QoL* in quantity. In this questionnaire, lower numbers report improvement in *QoL*. There is no heterogeneity between these studies (*p* = 0.96, $I^{2}=0\%$) and the overall result demonstrates a significant difference between melatonin and control groups (−5.95 [−9.54, −2.35], *p* = 0.001). Ultimately, the outcomes indicate the validity of *QoL* development in *HF* patients as the efficacy of melatonin (Figure 3).

#### 3.5.2 Effect of Melatonin on *EF*

*Garakyaraghi 2012* and *Hoseini 2022* studied the effect of melatonin on *EF* in patients with *HF*. The results of the studies were reported using the mean difference with 95% *CI*, *P*, and $I^{2}$ values. There is no significant heterogeneity between studies (*p* = 0.07, $I^{2}$ = 69%); Hence, these studies were approximately homogenous. Both *Garakyaraghi 2012* and *Hoseini 2022* reported development in EF due to oral melatonin (mean difference with 95% *CI*: 4.70 [0.97, 8.43] and 0.40 [−2.51, 3.31], respectively). The change of EF in the Hosseini 2022 study was not significant based on the confidence interval. Overall, a 2.39 [−1.82, 6.59] increase in *EF* was observed in the melatonin group compared to control. However, due to *p* = 0.27, the result of the analysis was insufficient to support a positive effect of melatonin on EF (Figure 3).

#### 3.5.3 Effect of Melatonin on *NYHA*

The effect of melatonin on *NYHA* change was studied just by the homogenous *Hoseini 2022* and *Garakyaraghi 2012* studies (*p* = 0.91 and $I^{2}$ = 0%). Odds ratio with 95% *CI*, *P*, and $I^{2}$ values were used for comparison of the intervention and control groups. In *Hoseini 2022* and *Garakyaraghi 2012* studies, 4.42 [0.47, 41.31] and 5.29 [0.57, 49.13] times improvement in *NYHA* class in the melatonin group to control was seen, respectively. Ultimately, 4.84 [1.00, 23.44] times development in *NYHA* class in melatonin to control group was observed. Furthermore, *p* = 0.05 demonstrated approximate validity in the global society (Figure 3).

### 3.6 Quality of Evidence

The effect of melatonin on *EF*, *QoL*, and *NYHA* in *HF* patients was investigated (Table 2). The certainty of evidence for *EF* and *NYHA* was low and *QoL* has moderate certainty of evidence. Each investigation was performed on more than 120 patients and all analyzed studies were *RCT*.

**Table 2:** Quality of evidence.

Outcome	Predicted absolute effects		Mean difference (first two) odds ratio (the last) with 95% *CI*	Number of participants	GRADE; Certainty of the evidence
	Improvement with melatonin	Improvement in control			
EF	—	—	2.39 [−1.82, 6.59]	124 (2 RCTs)	${⨁⨁◯◯}^{b,c}$
QoL	—	—	−5.95 [−9.54, −2.35]	120 (2 RCTs)	${⨁⨁⨁◯}^{b}\;$
NYHA	15 per 100	3 per 100	4.84 [1.00, 23.44]	124 (2 RCTs)	${⨁⨁◯◯}^{b,c}$

*Note:* Studies that related to the efficacy of melatonin on EF, QoL, and NYHA. Population: HF patients, Intervention: Oral melatonin, Comparison: Placebo tablets.

GRADE: Working Group Grades of Evidence

HIGH: There is complete confidence that the true effect is close to the estimate of the effect. ⨁⨁⨁⨁

MODERATE: The effect of the estimate is moderately confided: Despite the true effect is likely close to the effect estimate, it could be substantially different from the effect estimate. ⨁⨁⨁◯

LOW: The validity of the effect estimate is low: The true effect may be substantially different from the estimate of the effect. ⨁⨁◯◯

VERY LOW: Very little confidence exists in the effect estimate: The true effect is probably different from the effect estimate. ⨁◯◯◯

a) Low participant (one downgrade for imprecision)

b) Studies report data incompletely or incorrectly (one downgrade for risk of bias)

c) Studies missed more than 12.5% of participants to the outcome (one downgrade for risk of bias)

d) Studies were not RCT (one downgrade for risk of bias)

## 4 Discussion

This study is the first systematic review and meta‐analysis article that investigated and analyzed the effect of melatonin therapy on psychological, serum, and cardiovascular parameters in *HF* patients. Outcomes demonstrate melatonin has significant efficacy for improvement of patient's *QoL* (*p* < 0.05). However, the evidence does not support the positive effect of melatonin on *EF* and *NYHA* due to *p* ≥ 0.05. *NT‐Pro BNP*, *BP*, and *FMD* were investigated by *Hoseini'*s studies [22, 33] which reported the significant effect of melatonin on *NT‐Pro BNP* and *FMD* but the effect on *BP* was invalid. All selected studies for data analysis were *RCT*; in this way, three of them were published after 2020 [22, 23, 33]. Five studies were selected for data synthesis and analysis; the abstract of *Zaslavskaya's* study was published in PubMed database and its full‐text was published only in Russian and a domestic (Russian) journal; We can't find the article by texting emails to the first and corresponding authors so this article was extracted from data synthesis and analysis [35]. All remaining studies were performed in *I.R IRAN*, so it's needed to perform more studies in this context and also in other countries to determine the validity of the effect of melatonin on other parameters. The effect of melatonin on *HF* teenagers and children (under 18 years old) hasn't been studied till now, therefore medical society needs to conduct trials to show the effect of melatonin on under 18 years old patients. The other observed problem was missing the extreme number of participants compared to all patients in one study [34]. Compliance of patients to taking drugs could also affect the results of studies; fortunately, taking drugs by patients was checked in all selected studies. The side effects of melatonin in *HF* patients weren't recognized completely so more epidemic studies are needed to detect them. despite positive melatonin efficacy, it isn't prescribed by treatment staff so it is hoped that after publishing this article, an increase in the melatonin use for *HF* will be observed.

## 5 Conclusion

We conducted a systematic review and meta‐analysis to confirm the positive effect of melatonin on *HF* patients' development. Four articles were appropriate to protocol criteria from which three were used for data analysis. As a result of this study, melatonin therapy can be introduced as a new method for treating and palliating *HF* patients. After the melatonin intervention, a significant improvement in *QoL* was observed. However, no significant development in *EF* and *NYHA* class was observed in the final analysis. Furthermore, as effect of melatonin *NT‐Pro BNP* and fatigue were decreased but elevation of sleep quality, appetite, and *FMD* were seen.

## Conflicts of Interest

The authors declare no conflicts of interest.

## Data Availability

The data that support the findings of this study are available from the corresponding author upon reasonable request.
